# Cellular models and therapies for age-related macular degeneration

**DOI:** 10.1242/dmm.017236

**Published:** 2015-05-01

**Authors:** David L. Forest, Lincoln V. Johnson, Dennis O. Clegg

**Affiliations:** Molecular, Cellular, and Developmental Biology, University of California Santa Barbara, Santa Barbara, CA 93106-9625, USA

**Keywords:** AMD, RPE, Cell-culture models, hESC, iPSC, Stem-cell therapy

## Abstract

Age-related macular degeneration (AMD) is a complex neurodegenerative visual disorder that causes profound physical and psychosocial effects. Visual impairment in AMD is caused by the loss of retinal pigmented epithelium (RPE) cells and the light-sensitive photoreceptor cells that they support. There is currently no effective treatment for the most common form of this disease (dry AMD). A new approach to treating AMD involves the transplantation of RPE cells derived from either human embryonic or induced pluripotent stem cells. Multiple clinical trials are being initiated using a variety of cell therapies. Although many animal models are available for AMD research, most do not recapitulate all aspects of the disease, hampering progress. However, the use of cultured RPE cells in AMD research is well established and, indeed, some of the more recently described RPE-based models show promise for investigating the molecular mechanisms of AMD and for screening drug candidates. Here, we discuss innovative cell-culture models of AMD and emerging stem-cell-based therapies for the treatment of this vision-robbing disease.

## Abstract (precis)

**Summary:** Here, we discuss the emerging cell-culture models and potential stem-cell-based therapies for AMD, a blinding disorder that affects millions of people worldwide.

## Introduction

**Fig. 1.**
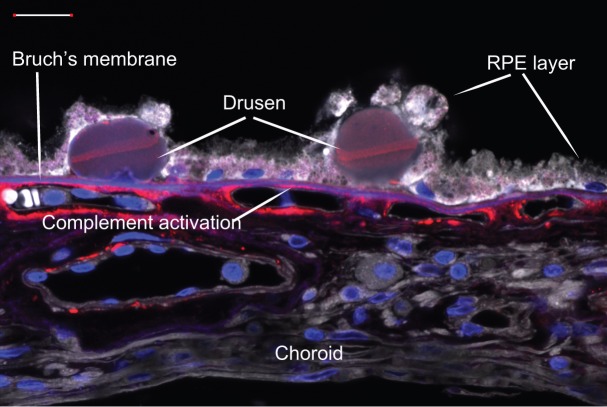
**Drusen deposits under RPE cells from an individual with AMD.** Confocal microscopy image of retinal tissue from an 82-year-old female individual with a history of age-related macular degeneration (AMD). Drusen deposits are implicated in the degeneration of retinal pigmented epithelial (RPE) cells. Here, a cell-membrane marker in gray shows degraded RPE cells overlying drusen. Areas of complement activation within the drusen and around the blood vessels are indicated in red by the terminal complement complex marker C5b-9. Nuclei are stained blue. Scale bar (upper left): 20 µm. Image credit: David L. Forest, UC Santa Barbara.

AMD – age-related macular degeneration – is a leading cause of blindness for millions of people over the age of 60. The disease is associated not only with visual impairment, but also with high rates of depression, anxiety and emotional distress (Berman and Brodaty, 2006). Visual dysfunction in AMD is associated with the degeneration of retinal pigmented epithelium (RPE) cells and of the light-sensing photoreceptor cells that they support. Degeneration of RPE cells in AMD seems to begin with impaired clearance of cellular waste material. This leads to a state of chronic inflammation in the eye, and eventually to the formation of abnormal deposits called drusen, which impair the function of RPE cells (Fig. 1).

Healthy adult RPE cells form a tightly interconnected sheet of cells positioned between the photoreceptors and a rich vascular layer, the choroid (or choriocapillaris). This arrangement creates a semi-permeable barrier that allows the RPE to selectively transport nutrients from the blood supply to the outer layers of the retina (Hewitt and Adler, 1989). Other important features of the RPE will be highlighted in the next section, which focuses on the pathology of AMD. The macula (or macula lutea – ‘yellow spot’) is a specialized anatomical feature of the retina that is responsible for focused, high-resolution color vision. A progressive loss of this fine-acuity central vision, due to loss of RPE cells and photoreceptors in the macula, is characteristic of AMD.

As discussed in more detail below, there are two main forms of the disorder: wet AMD, for which some treatment options exist (Table 1), and dry AMD, which is the most common form of the disease. Dry AMD is a candidate for emerging cellular transplantation therapies because there are currently no clinical treatments available. Several different cell types are being considered for therapeutic transplantation, including stem cells isolated from umbilical cord, neural progenitor cells, and RPE cells derived from pluripotent stem cells. Several of these therapies are currently in, or are rapidly approaching, clinical trials (Table 2). Pluripotent stem cells include human embryonic (hESCs) and induced pluripotent (iPSCs) stem cells. Both have the potential to become almost any cell type in the body (Thomson et al., 1998; Takahashi and Yamanaka, 2006). They also serve as model systems for the study of early eye developmental stages, and as source material for stem-cell-based therapies (Meyer et al., 2011).

**Table 1.**
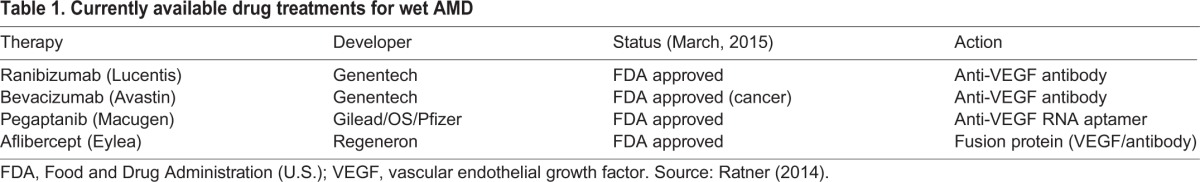
**Currently available drug treatments for wet AMD**

**Table 2.**
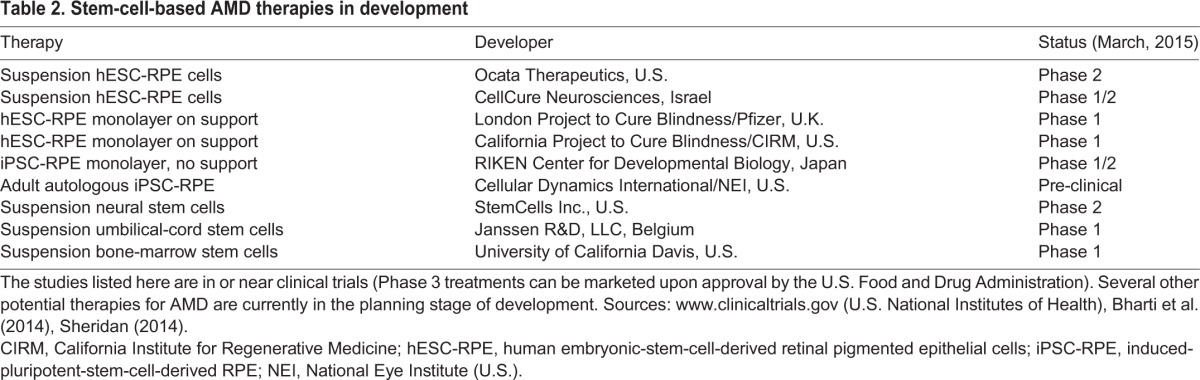
**Stem-cell-based AMD therapies in development**

In anticipation of their therapeutic use, multiple groups have derived RPE cells from both of these pluripotent cell types (Buchholz et al., 2009; Hirami et al., 2009; Kamao et al., 2014; Klimanskaya et al., 2004; Krohne et al., 2012). RPE cells derived from hESCs (hESC-RPE) are currently being used in clinical trials for macular degeneration. In these trials, a suspension of up to 150,000 cells was injected into an area between the degenerating photoreceptor cell and RPE cell layers (Schwartz et al., 2015). A recent follow-up study of the 18 patients involved in the trials revealed no serious safety issues related to the transplanted cells (Schwartz et al., 2015). Implantation of a single layer of stem-cell-derived RPE is another treatment approach currently under development (Carr et al., 2013; Idelson et al., 2009). Recently, a Japanese woman in her 70s was the first person to receive a transplanted layer of iPSC-RPE derived from her own skin cells (Cyranoski, 2014).

In this Review, we first summarize the main pathological features and disease mechanisms that are characteristic of AMD. Next, we introduce some of the innovative cell-based models in development. Finally, we discuss the potential of stem-cell-based therapies for the treatment of AMD. Animal models for AMD, although useful in some respects, fall short of recapitulating all aspects of the disease. An exhaustive summary of the many animal models currently in use is beyond the scope of this Review, which focuses on cell-culture models. More information on animal models of AMD can be found in one of the many papers on the subject (see Pennesi et al., 2012; Zeiss, 2010).

## The pathology of AMD

One way that RPE cells support visual function is by ingesting and disposing of photoreceptor cell outer segments. Outer segments are the site of phototransduction, where light energy is converted into an electrical signal. They are constantly produced by photoreceptor cells and the older segments are discarded as waste material on a daily cycle. The RPE cells internalize the old segments and recycle the light-sensitive molecule retinol back to the photoreceptors. Another crucial function of the RPE cells is to transport nutrients from the blood supply to the photoreceptors. As highlighted above and illustrated in Fig. 1, toxic deposits called drusen accumulate in the macula of individuals with AMD. Drusen are formed by cellular debris that is trapped between the single layer of RPE cells and Bruch's membrane, a specialized extracellular matrix (ECM) to which the RPE adheres (Fig. 1). The debris seems to act as a chronic inflammatory stimulus that initiates the process of drusen formation (Johnson et al., 2001). Drusen can eventually destroy RPE cells, and the resulting loss or disruption of RPE support functions consequently leads to photoreceptor degeneration. AMD primarily affects central vision, whereas some peripheral vision remains.

Advanced age is the primary risk factor for AMD. Physiological changes that generally occur past the age of 60 can impair cellular function in those at risk of the disease (Demontis et al., 2013). There are several other AMD-associated risk factors, which include genetic susceptibility, smoking and diet (McCarty et al., 2001). The disease typically manifests in two different forms that are identified as wet (exudative) or dry (atrophic) AMD.

Wet AMD is currently treated with ocular injections that delay the abnormal growth of blood vessels into the retina, which characterizes this form of the disease. Current wet AMD drug treatments focus on inhibiting vascular endothelial growth factor (VEGF) (Table 1), which stimulates blood vessel production. Recently, the possibility of adverse side effects due to ocular administration of VEGF has received attention. A specific role for VEGF in the regulation of RPE function and the long-term effects of anti-VEGF treatments in humans are unknown (Ablonczy et al., 2011). However, in mouse models, prolonged treatment with anti-VEGF therapy correlates with increased death of photoreceptors and their supporting cells within the retina (Ford et al., 2012; Saint-Geniez et al., 2008).

Most of those afflicted with advanced AMD have the dry form of the disease, which is currently untreatable. In this form, the disease frequently reaches an end-stage condition called geographic atrophy (GA). GA is characterized by a progressive loss of photoreceptor cells, RPE cells and the underlying blood vessels (Sunness, 1999). GA begins with small, focal lesions that typically form around the macula and eventually coalesce into a large area of atrophy. This process is initially perceived as tunnel vision that, over a period of years, eventually consumes central vision.

Impaired immune-system regulation might contribute to the progression of AMD. In particular, disruption of the complement system is implicated in AMD development (Anderson et al., 2010). The complement system is a related group of proteins that circulate in the bloodstream and form an integral part of the immune system. When activated, complement proteins are largely responsible for pathogen recognition and removal. Complement activation also initiates an inflammatory response at sites of injury or infection. Variations in several complement-system genes are associated with AMD, some of which might cause the complement system to be overactive, resulting in a chronic inflammatory condition (Kawa et al., 2014; Hageman et al., 2005). This abnormal inflammatory stimulus adversely affects RPE cells and promotes drusen formation (Jakobsdottir et al., 2008) (Fig. 1). However, the exact mechanism by which complement-system abnormalities contributes to AMD development has not been established (Yates et al., 2007). Several new drug compounds that target the complement system are in development (Table 3), but are not currently approved for clinical use (Ambati et al., 2013). Both the wet and dry forms of AMD could be treated by these newly emerging anti-complement therapies (Rohrer et al., 2010).

**Table 3.**
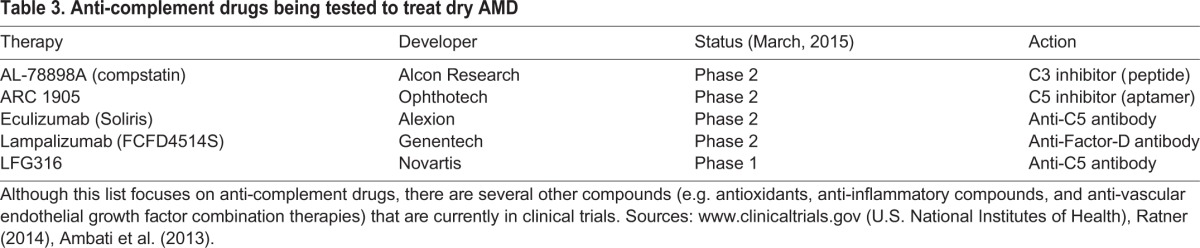
**Anti-complement drugs being tested to treat dry AMD**

In summary, AMD is a complex disorder that involves the interaction of genetic and environmental factors, all combined with the unique anatomy of the human macula (Pennesi et al., 2012). Compromised RPE function eventually leads to photoreceptor cell death and partial blindness that affects central vision. There are some treatment options for wet AMD, but currently none for dry AMD. Next, we discuss some of the promising new cell-based disease models of AMD that are currently in development.

## Cell-based models of AMD

Because of the complexities of AMD and the unique features of the human eye, animal models, although useful in some respects, fall short of recapitulating all aspects of AMD (Zeiss, 2010). Cell-culture models are important tools used to study the physiology and pathology of cells and tissues, including RPE cells (Maminishkis et al., 2006; Pfeffer, 1991). Advances in our understanding of AMD biology, improved cell-culture systems, and the availability of stem-cell technologies offer great potential for modeling the salient features of this disease. The goal of cell-based disease modeling is to relate changes observed in cultured cells to physiologically relevant changes in the organism (Carlson et al., 2013). Merely obtaining a desired phenotype in culture does not guarantee that it is of physiological relevance to disease (Cristofalo and Pignolo, 1995). However, innovative new methods and technologies are rapidly improving the quality and utility of cell-based AMD models.

Cell-culture models are advantageous because they are defined systems in which experimental conditions can be controlled and manipulated. Also, the results are usually more reproducible than those from animal models (Hornof et al., 2005). Primary cultures of human fetal RPE (hfRPE) are particularly effective tools in AMD research because they closely model the function and metabolic activity of native RPE (Ablonczy et al., 2011). Thus, they have become a standard to which other RPE cell types are compared (Adijanto and Philip, 2014). Other RPE cell types used in AMD research include RPE derived from stem cells and the immortalized ARPE-19 cell line (Dunn et al., 1996).

*In vivo*, the apical RPE surface interfaces with retinal photoreceptors and its basal surface attaches to Bruch's membrane, a specialized structure composed of collagen, laminin, elastin and fibronectin (Booij et al., 2010). This porous ECM allows for selective metabolite exchange to occur between the retina and its primary blood supply, the choriocapillaris (Curcio and Johnson, 2013). Cultured RPE cells require similar substrate attachment to attain differentiated structure and function. As such, the ECM that lies beneath the RPE is critical to cell-based models and therapies. For example, purified ECM proteins such as collagen IV, laminin and vitronectin differentially influence hESC-RPE growth, pigmentation and barrier function (Sorkio et al., 2014; Williams and Burke, 1990). The use of purified ECM proteins also improves the production of differentiated iPSC-RPE cells (Rowland et al., 2013).

Bioengineered polymer support matrices also improve stem-cell survival and differentiation (Enzmann et al., 2009). In addition, support matrices promote the formation of a single layer of polarized RPE cells with specialized apical and basal features. Disruption of this normal polarized configuration of RPE cells is implicated in retinal disease (Nasonkin et al., 2013). Supporting membranes and polarized RPE monolayers are used in drug testing and to analyze molecular transport and secretion (Sonoda et al., 2009).

Cell-based models also enable the experimental formation of sub-RPE deposits (Amin et al., 2004). RPE cells grown on porous supports form subcellular deposits that contain drusen-associated molecules and activated complement proteins when exposed to human serum (Johnson et al., 2011). This model system was recently used as an experimental platform to test new peptide-based complement-system inhibitors (Gorham et al., 2013). The model also demonstrated that RPE cells with a defective version of Factor-H, a complement-system regulator protein, are more susceptible to complement attack when exposed to a potentially toxic metabolite from photoreceptor outer segments (Radu et al., 2014).

Autologous, patient-derived, iPSCs can be used to generate RPE cells for disease modeling, personalized medicine and patient-specific drug discovery (Mack et al., 2014; Jin et al., 2011). Thus, an AMD patient's own cells could be used to create a more accurate model of their disease (Phillips et al., 2014; Wahlin et al., 2014). This approach was recently used to model vitelliform macular degeneration, an early-onset form of Best disease (Singh et al., 2013). A similar patient-derived iPSC-RPE model revealed that AMD-associated gene variants [namely, of the age-related maculopathy susceptibility 2 (*ARMS2*) and the high-temperature requirement factor A1 (*HTRA1*) genes] disrupted the normal antioxidant function of the cells (Yang et al., 2014). One limitation of iPSC-based approaches is that AMD might involve systemic defects that are not recapitulated in the culture of a single cell type. More complex human tissue structures that better reproduce physiological conditions and disease characteristics are being developed for future research (Gamm et al., 2013). By combining an RPE monolayer with other retinal cells, or with a modeled choroid capillary bed, investigators could study the interaction between cells that might contribute to disease.

In the human eye, the blood–retinal barrier consists of three layers: the RPE monolayer, the Bruch's membrane and the underlying choriocapillaris (a network of small blood vessels). Recreating this native architecture should lead to more physiologically relevant models (Lehr, 2005). A functional-barrier model was created with cultured RPE and human vascular cells separated by amniotic membrane (Hamilton and Leach, 2011). Synthetic Bruch's membranes constructed from fibroin, a silk protein, also support the co-cultivation of RPE cells and microvascular endothelial cells (Shadforth et al., 2012). Such three-dimensional cell-culture systems could be used to model the development of wet AMD by recreating disease-associated interactions among vascular cells, Bruch's membrane and RPE cells (Feigl and Hutmacher, 2013). Stem-cell-based three-dimensional models should also greatly accelerate AMD research and drug development (Pampaloni et al., 2007).

Future models and therapies might also take advantage of layered retinal tissues that spontaneously form in stem-cell cultures (Westenskow et al., 2014). A structure similar to the embryonic optic cup, which contains integrated RPE and neurosensory layers, can self-organize in mouse and human stem-cell cultures (Zhong et al., 2014; Eiraku and Sasai, 2012). If this process could be controlled, such engineered tissues might be used as a clinical-grade transplant to replace entire sections of damaged retina (Nakano et al., 2012). In the following section, we further investigate the cellular transplantation strategies currently in development as potential AMD therapeutics.

## Cell-based therapies for AMD

**Fig. 2.**
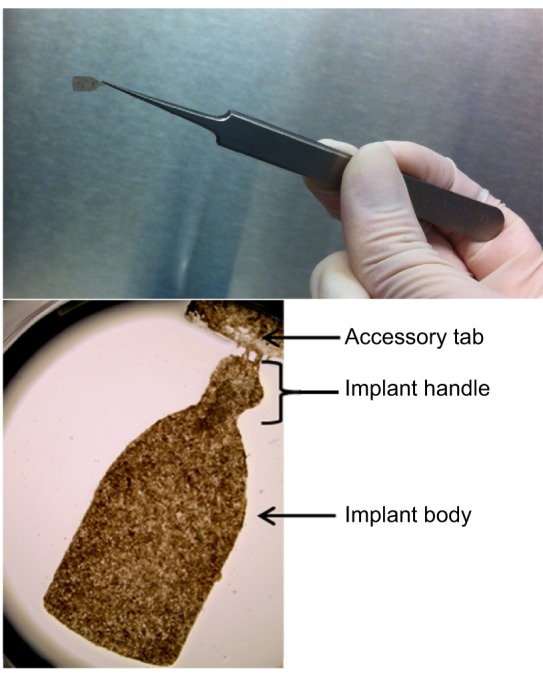
**An RPE cell implant.** A patch of therapeutic RPE cells that could be implanted to treat those afflicted with age-related macular degeneration (AMD). These hESC-RPE cells were grown on a parylene support (the implant body measures approximately 3.5×6 mm). Image credits: Dr Britney Pennington, UC Santa Barbara (upper panel), University of Southern California (lower panel).

RPE cell transplantation is a promising clinical strategy for treating AMD (Carr et al., 2013; Rowland et al., 2013). Both hESC-RPE and human iPSC-RPE are currently in clinical trials for AMD (Cyranoski, 2014; Schwartz et al., 2015) (Table 2). There are two main strategies for cell transplantation: (1) injection of a suspension of cells and (2) surgical implantation of an RPE monolayer, with or without a supporting membrane (Fig. 2). In the Royal College of Surgeons (RCS) rat, a classic animal model of retinal degeneration, injected hESC-RPE cell suspensions showed some incorporation into the native RPE layer and restored visual function (Lu et al., 2009). Long-term studies of the injected cells in mice showed no tumor formation over the lifetime of immune-system-deficient mice (Lu et al., 2009). Although the injection procedure is less invasive than monolayer implantation, injected RPE cells tend to form clusters and show limited phagocytosis of photoreceptor outer segments in rat models (Carr et al., 2009). An experiment comparing injection and implantation of hESC-RPE revealed that implanted monolayers survived longer (for at least 12 months) without evidence of tumor formation in immunocompromised rats (Diniz et al., 2013).

Initial results from the first human clinical trials using suspension injections of hESC-RPE indicated that patients showed no signs of tumor formation (Schwartz et al., 2012). A more recent follow-up study of the 18 patients also revealed no serious safety issues related to the injected cells (Schwartz et al., 2015). Although a trend towards improved vision was noted, the trial was designed to assess safety. More extensive trials will be needed to determine meaningful efficacy. A scaffold-free layer of iPSC-RPE, designed for clinical use, also showed no immune rejection or tumor formation when implanted in a primate model (Kamao et al., 2014). Based on this research, a Japanese woman recently became the first individual with AMD to ever receive a transplanted layer of autologous iPSC-RPE cells (Cyranoski, 2014). Patient-derived cells minimize the risk of immune rejection and could eventually be used for gene therapy. In this case, the iPSCs would be altered to remove a risk-conferring gene variant. The cells could then be differentiated into healthy RPE cells and transplanted into the retina (Selvaraj et al., 2010). A proof-of-concept experiment demonstrated that disease-free blood-cell progenitors could be created from individuals with Fanconi anemia (Raya et al., 2009).

Multipotent stem cells that reside within the adult retina, called RPE stem cells (RPESCs), might be another cell source for replacement therapy or disease modeling (Salero et al., 2012). When exposed to chronic oxidative stress in culture, these cells upregulate expression of several drusen-related proteins, demonstrating their use as a model for early AMD (Rabin et al., 2013). Because adult RPESCs retain the ability to proliferate, they could be a source of therapeutic cells to repair damaged RPE monolayers (Coffey, 2012). A polarized RPESC monolayer, grown on a biocompatible support membrane, retained RPE characteristics for over a month after sub-retinal implantation in a rabbit model of AMD (Stanzel et al., 2014).

Future RPE implants might include biocompatible scaffolds that mimic a healthy Bruch's membrane (Liu et al., 2014). For example, nanopatterned, porous poly(ε-caprolactone) (PCL) films are biocompatible, allow for metabolite transport and improve hfRPE cell function compared with non-porous PCL or porous polyester (McHugh et al., 2014). There are many other polymers and engineered materials with potential uses in RPE transplantation, including parylene (Croze and Clegg, 2014).

Parylene, a xylene-based hydrocarbon polymer that is already approved for biomedical use, can be engineered with ultrathin regions that have permeability similar to that of Bruch's membrane (Lu et al., 2012). hESC-RPE cultured on these ultrathin parylene-C membranes are able to adhere, proliferate, develop polarized monolayers and maintain RPE characteristics (Lu et al., 2012). Innovative surgical techniques were developed to implant the parylene substrates into a rat model of AMD, where >98% of the transplanted RPE cells survived the procedure (Hu et al., 2012).

## Conclusions and future directions

There is now considerable basic research and private-sector interest in producing RPE cells for transplantation. Mass production of differentiated cells with the functional morphology and characteristic marker expression of RPE cells is now possible (Maruotti et al., 2013). However, potential immune responses and transplant rejection remains a significant challenge for cellular therapies. Advanced methods to create genetically matched cell lines, such as somatic cell nuclear transfer, remain to be developed (Yabut and Bernstein, 2011). However, it is feasible to create and maintain banks of ‘super donor’ hESC-RPE cell lines to minimize transplant rejection and the need for immunosuppressive drugs (Lund et al., 2006). It is also possible to create a bank of pluripotent cell lines to match the human leukocyte antigen ‘cell type’ and minimize immune reactions for a large percentage of the population (Nakatsuji et al., 2008).

The optimal transplant strategy for long-term RPE survival and function remains to be determined (Kvanta and Grudzinska, 2014). In addition, some questions remain regarding the safety and efficacy of differentiated cells derived from iPSCs (Kokkinaki et al., 2011). Recent studies show that iPSCs are prone to genetic and epigenetic abnormalities relative to the progenitor cell, early-passage iPSCs or hESCs, with a higher number of mutations, copy number variations (CNVs) and unusual DNA methylation patterns (Pera, 2011). DNA sequencing analysis of 20 human iPSC lines revealed that some of those variations were already present in the donor cells (Abyzov et al., 2012). In other words, there might be a background level of cellular ‘mosaicism’ in donor tissues that can lead to cell-line variability. New analytical tools are being developed to provide genome-wide reference maps of DNA methylation and gene expression profiles for multiple stem-cell lines. For example, these tools were used to assess the epigenetic and transcriptional similarity of 32 different hESC and iPSC lines (Bock et al., 2011). This could lead to a comprehensive method to characterize the genetic features of any stem-cell line, and to predict their differentiation efficiencies.

RPE-cell-replacement therapies are of great potential and offer considerable hope for the treatment of AMD. However, stem-cell-based disease modeling and transplantation requires a long-term, multi-disciplinary approach. This coordinated effort must integrate biomedical research and materials science, together with clinical application, commercial interest and financing. Therefore, significant challenges, and opportunities, remain in order to fully develop these therapies.
